# Supplementary material for: Applying a deep learning pipeline to classify land cover from low-quality historical RGB imagery
Source: PeerJ Comput Sci. 2024 May 14;10:e2003. doi: 10.7717/peerj-cs.2003 (PMC11157591; doi:10.7717/peerj-cs.2003)
Supplement: Supplemental Information 1 [file peerj-cs-10-2003-s001.pdf]

Supporting Information for: Applying a deep learning pipeline to classify low-quality historical RGB imagery into land cover classes

Harold N. Eyster[1,2,*] and Brian Beckage[1,2]

[1]Gund Institute for Environment, University of Vermont, USA
[2]Department of Plant Biology, University of Vermont, USA

*Corresponding author. Please direct any questions or comments to haroldeyster@gmail.com.

1 Additional figures

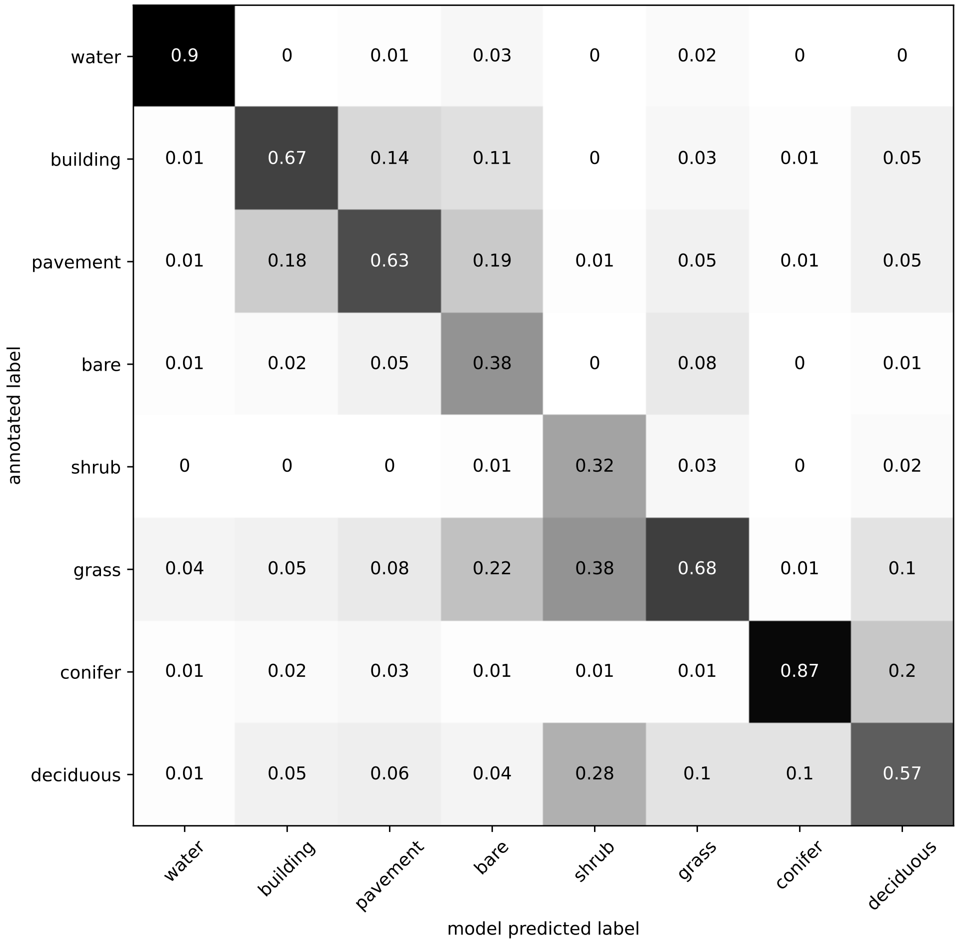

Figure S1: Modern confusion matrix, normalized by column.

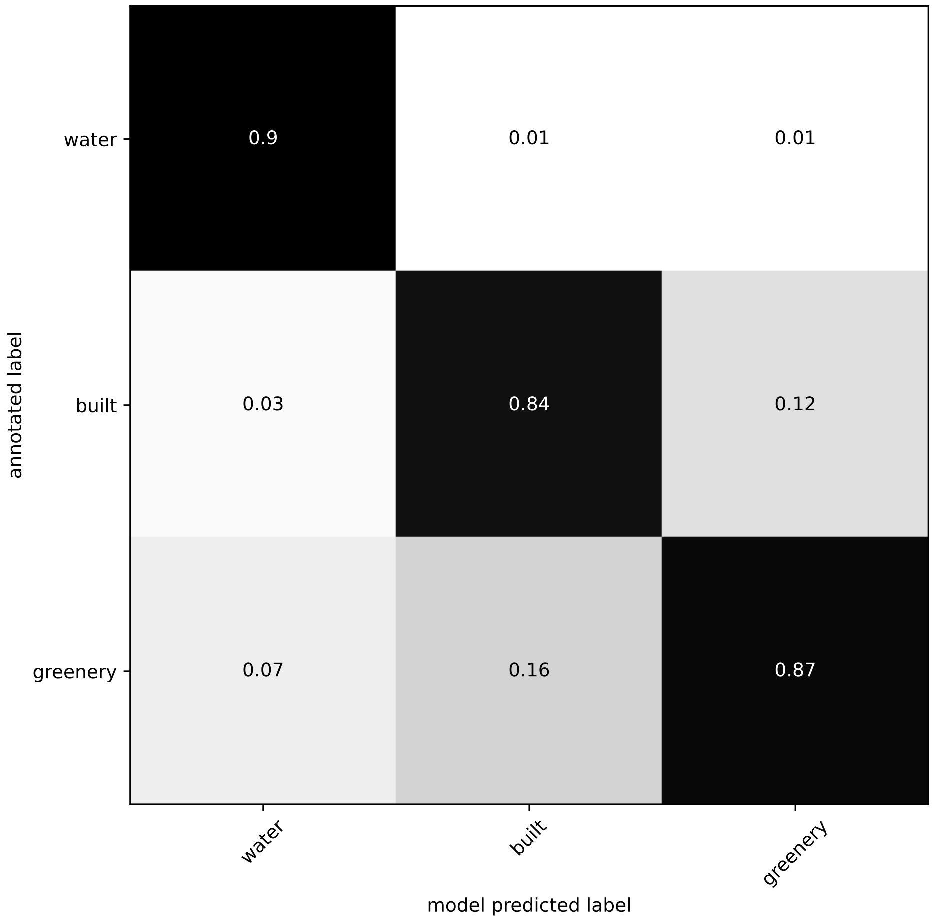

Figure S2: Modern confusion matrix after binning into water, built, and greenery categories, normalized by column.

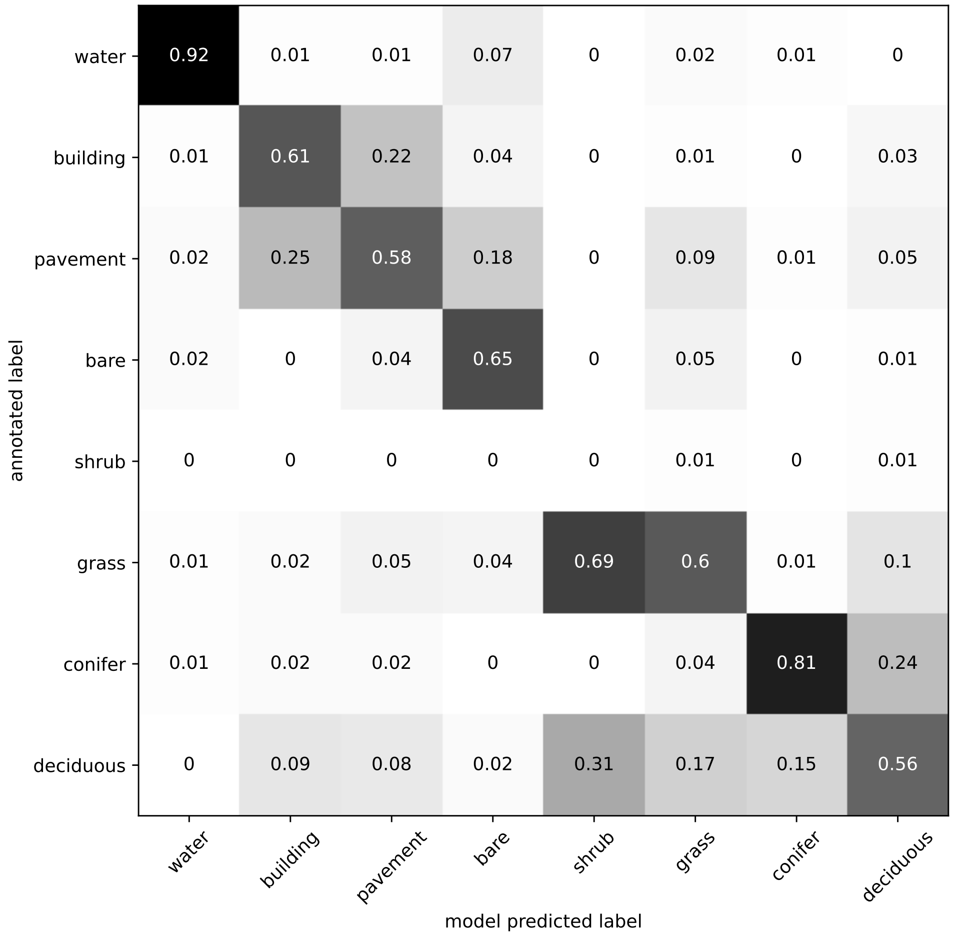

Figure S3: Historical confusion matrix after training on hand-annotated historical imagery augmented by rotation, normalized by column.

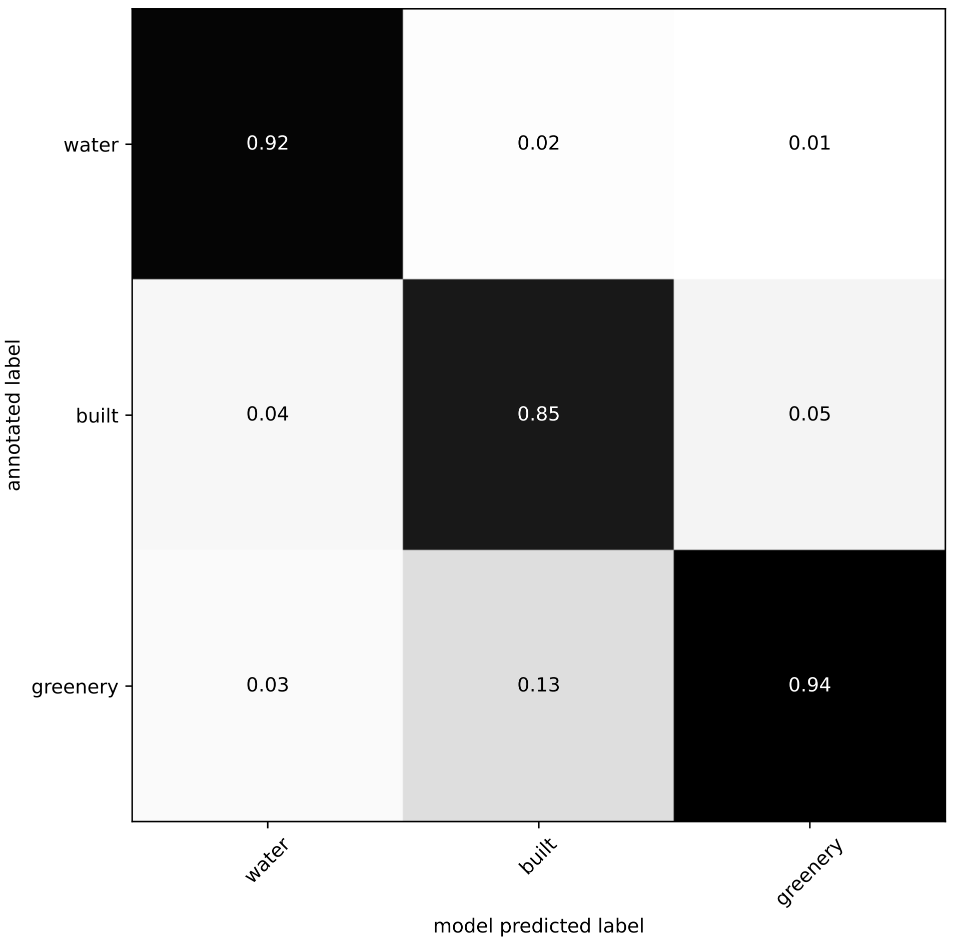

Figure S4: Historical confusion matrix after binning into water, built, and greenery categories and after training on hand-annotated historical imagery augmented by rotation, normalized by column.

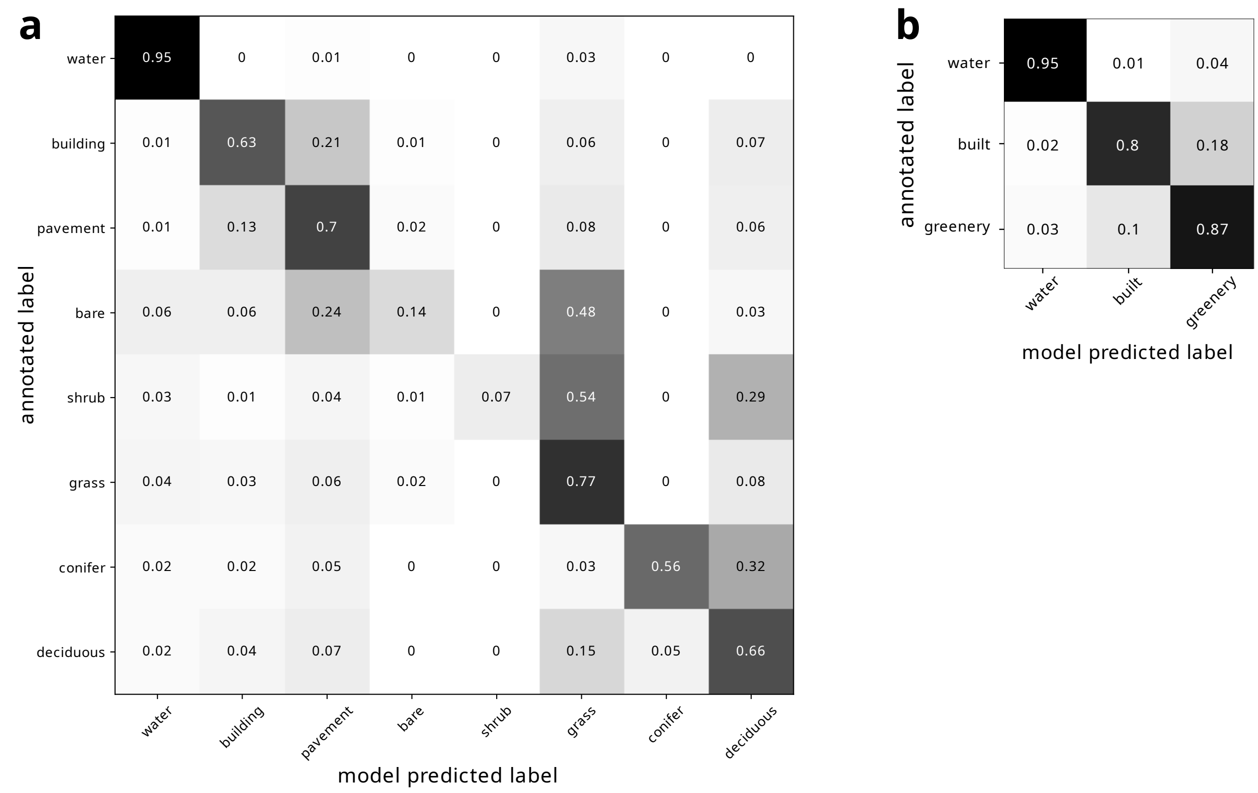

Figure S5: Confusion matrix of modern land cover model predictions, normalized by row of (a) all land cover types and (b) binned land cover categories

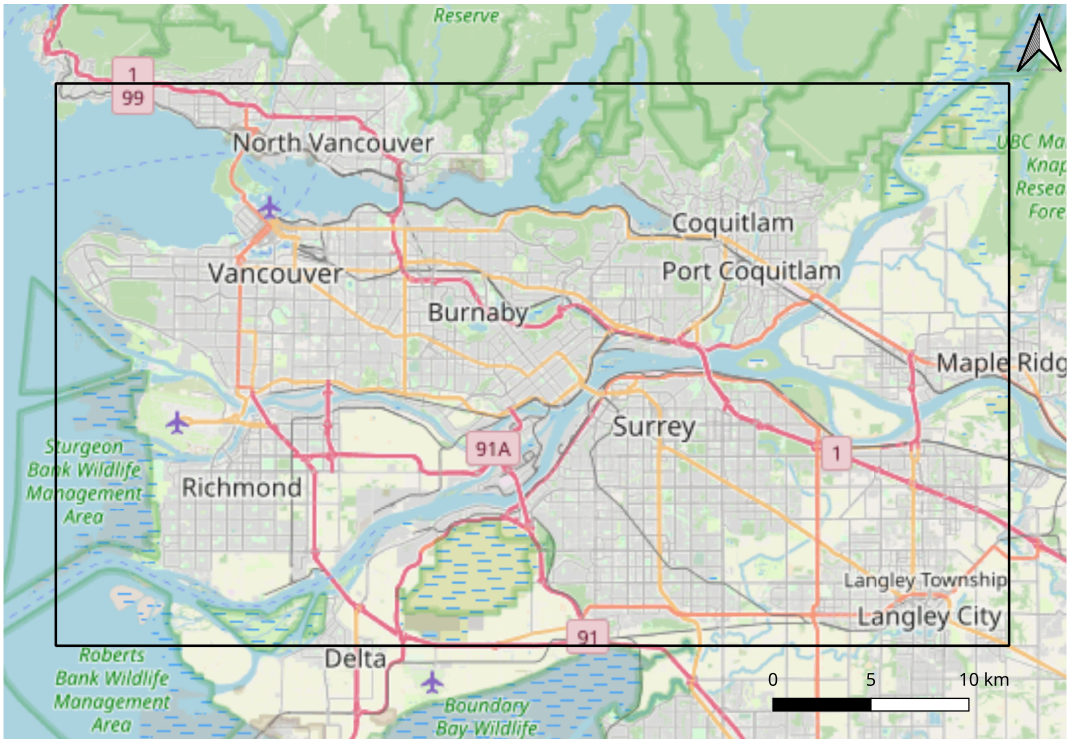

Figure S6: Map of Metro Vancovuer, with black bounding box showing extent of training data. Basemap © OpenStreetMap.

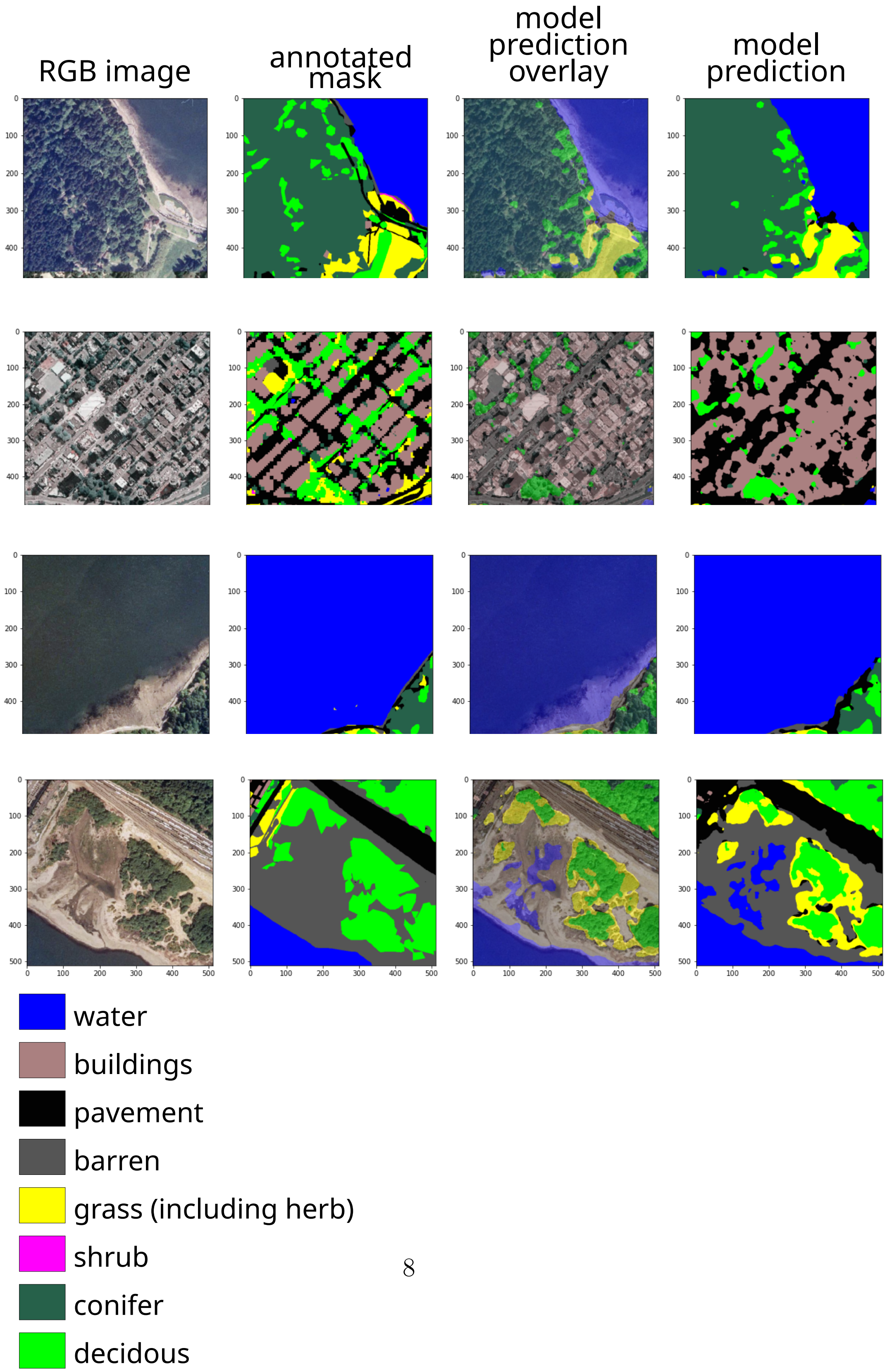

Figure S7: Historical RGB imagery tiles and land cover predictions.
